# Effect of lung pericyte-like cell ablation on the bleomycin model of injury and repair

**DOI:** 10.1152/ajplung.00392.2021

**Published:** 2022-02-23

**Authors:** Chi F. Hung, Carole L. Wilson, Yu-Hua Chow, Wayne C. Liles, Sina A. Gharib, William A. Altemeier, Lynn M. Schnapp

**Affiliations:** ^1^Division of Pulmonary, Critical Care, and Sleep Medicine, Department of Medicine, University of Washington, Seattle, Washington; ^2^Center for Lung Biology, University of Washington, Seattle, Washington; ^3^Division of Allergy, Pulmonary, and Critical Care Medicine, Department of Medicine, School of Medicine and Public Health, University of Wisconsin, Madison, Wisconsin; ^4^Division of Allergy and Infectious Diseases, Department of Medicine, University of Washington, Seattle, Washington

**Keywords:** bleomcyin, fibrosis, inflammation, lung pericytes, myofibroblasts

## Abstract

We previously showed that pericyte-like cells derived from the FoxD1-lineage contribute to myofibroblasts following bleomycin-induced lung injury. However, their functional significance in lung fibrosis remains unknown. In this study, we used a model of lung pericyte-like cell ablation to test the hypothesis that pericyte-like cell ablation attenuates lung fibrosis in bleomycin-induced lung injury. Lung fibrosis was induced by intratracheal instillation of bleomycin. To ablate pericyte-like cells in the lung, diphtheria toxin (DT) was administered to *Foxd1-Cre;Rosa26-iDTR* mice at two different phases of bleomycin-induced lung injury. For early ablation, we coadministered bleomycin with DT and harvested mice at *days 7* and *21*. To test the effect of ablation after acute injury, we delivered DT 7 days after bleomycin administration. We assessed fibrosis by lung hydroxyproline content and semiquantitative analysis of picrosirius red staining. We performed bronchoalveolar lavage to determine cell count and differential. We also interrogated mRNA expression of fibrosis-related genes in whole lung RNA. Compared with DT-insensitive littermates where pericyte-like cells were not ablated, DT-sensitive animals exhibited no difference in fibrosis at *day 21* both in the early and late pericyte ablation models. However, early ablation of pericytes reduced acute lung inflammation, as indicated by decreased inflammatory cells. Our data confirm a role for pericytes in regulating pulmonary inflammation in early lung injury.

## INTRODUCTION

Cells of different lineages differentiate into myofibroblasts during lung injury, but the functional contribution of specific myofibroblast precursors to lung fibrosis remains controversial (1–7). Activated myofibroblasts display enhanced matrix invasion, resistance to apoptosis, and enhanced matrix deposition. Their persistence ultimately leads to replacement of normal alveolar architecture with scar tissue that is rich in extracellular matrix. Studies characterizing lung myofibroblasts in human pulmonary fibrosis and experimental models using lineage-tracing identified potential myofibroblast progenitors including resident fibroblasts, circulating bone-marrow-derived progenitors (i.e., fibrocytes), epithelial cells, and more recently, pericytes (8). However, the functional contribution of these progenitors to the development of lung scar tissue remains poorly defined. In this study, we sought to define the functional contribution of pericytes to lung fibrosis in the bleomycin model of lung injury.

Lung pericytes are perivascular stromal cells that reside adjacent to endothelial cells in the microvascular niche. They are critical in the development of vasculature during embryonic development and play a role in maintaining endothelial integrity during homeostasis in a number of organs (9). Recent work by our group demonstrated a role for pericytes as immune regulators in the lung (10, 11). The role of pericytes in lung fibrosis remains a much-debated issue. There is conflicting evidence regarding the contribution of pericytes to myofibroblasts, the key effector cells responsible for the histopathologic features in fibrosis (4, 5, 12). We previously demonstrated that up to 60% of lung myofibroblasts were derived from FoxD1-derived pericytes in a model of bleomycin-induced fibrosis (12). However, the clinical significance of this finding is unclear.

We developed a model of lung pericyte ablation by oropharyngeal aspiration of diphtheria toxin (DT) to genetically susceptible mice (13). In this follow-up study, we leveraged the lung pericyte ablation model to determine whether pericytes functionally contribute to lung fibrosis, as defined by collagen I deposition, in the bleomycin model of lung injury.

## METHODS

### Animals and Reagents

The University of Washington (UW) and Medical University of South Carolina (MUSC) Institutional Animal Care and Use Committees approved all experiments involving the use of mice described in this report. For ablation studies, bitransgenic mice expressing Cre-recombinase under the *FoxD1* promoter and simian diphtheria toxin receptor (iDTR) under the *Rosa26* promoter (*Foxd1-Cre;Rosa26-iDTR*) were generated as previously described (13). Male and female transgenic mice between ages of 8–12 wk and at least 16 g in body weight were included in animal experiments. Investigators were blinded to genotypes of animals until after harvesting and processing of samples for analysis. For microarray studies, *Foxd1-Cre;Rosa26-tdTomato* mice were bred with Coll-GFP transgenic mice (12). Bleomycin (SICOR Pharmaceuticals, Inc., Irvine, CA or TEVA) in lyophilized form was obtained from the UW Medical Center and MUSC pharmacies. Diphtheria toxin was obtained from Sigma-Aldrich (Cat. No. D0564).

#### Lung cell sorting and analysis of gene expression by microarray.

At *day 7* following bleomycin administration, lung tissue underwent enzymatic digestion and cell sorting as previously described with minor modifications (10). We focused on the following cell populations for sorting: FoxD1-derived^+^/Coll-GFP^–^ pericytes (Peri), FoxD1-derived^+^/Coll-GFP^+^ pericytes (Peri^Fibro^), and FoxD1-derived^–^/Coll-GFP^+^ stromal fibroblasts (Fibro). Following RNA isolation of sorted GFP^+^ and/or tdTomato^+^ cells (Qiagen Micro RNeasy kit per manufacturer protocol), RNA integrity was confirmed with an Agilent 4200 TapeStation (Agilent Technologies, Inc., Santa Clara, CA). Samples were labeled and randomly hybridized to two MouseWG-6 v2.0 Expression BeadChip microarrays (Illumina, Inc., San Diego, CA) at the Genomics Shared Resource Core of the University of Texas Southwestern. Each MouseWG-6 v2.0 platform enables the interrogation of six samples in parallel and comprises ∼45,000 well-annotated murine transcripts.

#### Microarray analysis.

Probe intensities underwent variance stabilization and quantile normalization using the Bioconductor package *lumi* (14). Detailed microarray information and raw data meeting MIAME requirements have been deposited at Gene Expression Omnibus (https://www.ncbi.nlm.nih.gov/geo/, GSE184761). Multidimensional scaling using principal component analysis (PCA, Partek Flow, St. Louis, MO) was performed and used to identify and remove two outlier samples with poor hybridization. Differentially expressed genes (DEGs) were identified using a Bayesian implementation of the parametric *t* test (15, 16) coupled with false-discovery rate (FDR) analysis using Benjamini–Hochberg’s method (17). An adjusted *P* value cutoff <0.05 and a twofold change was used as the significance threshold. Gene Set Enrichment Analysis (GSEA) was applied to the entire microarray data (18). We focused this analysis on well-curated pathways from the Molecular Signatures Database (MSigDB v7.4) that included 50 “Hallmark” gene sets and ∼2,750 “canonical pathway” gene sets (KEGG, Reactome, Pathway Interaction Database, Biocarta, etc.). Enriched gene sets were identified using an FDR ≤ 0.05 and grouped into larger biological modules based on functional overlap. For both the differential gene expression analysis and GSEA, we compared the three cell populations (Peri, Peri^Fibro^, Fibro) with each other at the uninjured baseline time point and at *day 7* following bleomycin injury. For each cell population, we also compared baseline versus bleomycin. Gene product relationships between differentially upregulated pericyte (Peri) genes in bleomycin injury versus control conditions were investigated using Ingenuity Pathway Analysis (IPA), and depicted as an interacting network.

### Pericyte-Like Cell Ablation and Lung Injury Model

#### Bleomycin-induced lung injury, early ablation.

For coadministration of bleomycin and diphtheria toxin, bleomycin and diphtheria toxin were first reconstituted in sterile saline to a concentration of 0.4 U/mL (bleomycin) and 0.25 ng/µL (DT). Bleomcyin (0.8 U/kg mouse body wt) and diphtheria toxin (0.5 ng/g mouse body wt) were then coadministered to mice by intratracheal instillation (2 µL/g mouse body wt) under isoflurane anesthesia as previously described (19). Mice were monitored per protocol and harvested at *days 7* and *21*.

#### Bleomycin-induced lung injury, late ablation.

For sequential administration of bleomycin (*day 0*) followed by DT (*day 7*), bleomycin was reconstituted to a concentration of 1.3 U/mL and delivered intratracheally under isofluorane anesthesia as previous described (19). We induced ablation by administering DT (0.5 ng/g mouse body weight) via oropharyngeal aspiration under isoflurane anesthesia as previously described (13). Flow cytometry to confirm ablation efficiency 7 days after DT was performed as previously described (13).

#### Protein assay.

Protein measurements of bronchoalveolar lavage fluid (BALF) collected from animals were performed using Bradford Protein Assay (Bio-Rad), as per the manufacturer’s instructions.

#### Cell differential.

Nucleated cells in BALF were stained with Diff-Quik, and 300 cells/sample were manually counted under microscopy.

#### Hydroxyproline assay.

Left lungs from mice were collected at *day 21* following bleomycin administration. Left lungs were weighed and homogenized in 1 mL deionized water. Hundred microliters of lung homogenate was mixed in 100 µL of 37% hydrochloric acid (Sigma, Cat. No. 258148, St. Louis, MO) and heated at 120°C for 3 h. Fifty microliters of the acid hydrolyzed samples was added to 96-well flat bottom microplates in duplicates. Hydroxyproline content was then measured using the Hydroxyproline Assay Kit (Sigma, Cat. No. MAK0008, St. Louis, MO), as per the manufacturer’s protocol. Total protein content in the left lung homogenate was obtained by mixing 100 µL of lung homogenate with 100 µL of 2× protein lysis buffer. The sample was mixed and kept on ice for 30 min, with vortexing every 10 min. The samples were spun down and the supernatant was collected. Protein content in the protein lysate supernatant was detected by standard colorimetric assay using the Pierce BCA Protein Assay Kit (Thermo Fisher Scientific, Cat. No. 23225, Rockford, IL). Hydroxyproline measurements were then normalized to total protein content (expressed as micrograms of hydroxyproline/left lung).

#### Real-time PCR.

Total RNA was isolated from homogenized lung tissue using NucleoSpin RNA kit (Macherey–Nagel) in conjunction with DNase treatment as per the manufacturers’ specifications. Total RNA was reverse-transcribed to cDNA using Applied Biosystems High-Capacity cDNA Archive Kit or iScript Reverse Transcription SuperMix (Bio-Rad, Hercules, CA). Real-time PCR was done using an ABI 7900HT instrument with the use of predesigned primers and probes (ABI TaqMan Gene Expression Assays and IDT PrimeTime Std qPCR Assays). Quantification of gene expression was normalized to *Hprt* (endogenous controls). Predesigned primers and probes include *Acta2* (Mm01546133_m1, ABI), *Col1a1* (Mm00801666_g1, ABI), *Fn1* (Mm01256744_m1, ABI), *Hprt* (Mm03024075_m1, ABI), *Ccl2* (Mm.PT.58.42151692, IDT), *Ccl7* (Mm.PT.58.17719534, IDT), *Ccl20* (Mm.PT.58.12466372, IDT), *Cxcl1* (Mm.PT.58.42076891, IDT), *Cxcl2* (Mm.PT.58.10456839, IDT), *Cxcl10* (Mm.PT.58.435758.27, IDT), *Il-6* (Mm.PT.58.10005566, IDT), and *Tnf* (Mm.PT.58.12575861, IDT). Analysis was performed in MS Excel calculating RQ by 2^−^Δ^ΔCT^ method. Gene expression level was normalized to averaged value for Cre^−^ group.

#### Histology.

Right lungs were inflated with 10% neutral buffered formalin (VWR) at 25 cmH_2_O pressure and fixed for 24 h at room temperature. Lung sections (4 µm) prepared from formalin-fixed paraffin-embedded (FFPE) tissue blocks were stained with hematoxylin and eosin (H&E) for histology, and with picrosirius red/fast green for histological evaluation of collagen deposition (Histology and Imaging Core at the University of Washington, Seattle, WA). Stained whole slides were digitized (Hamamatsu NanoZoomer), and analysis of collagen-to-total lung area was performed on picrosirius-stained sections following masking of collagen-staining around central airways and vasculature (Viziopharm). Immunohistochemical staining of PDGFRβ on FFPE lung sections was automated using the Leica BOND immunostainer (Leica Biosystems). Briefly, slides were deparaffinized, followed by antigen retrieval with citrate solution for 20 min at 100°C. Slides were then blocked with Leica Bond Peroxide block for 5 min at room temperature (RT). Following blocking, lung sections were incubated with rabbit anti-PDGFRβ antibody (Millipore, 04–397) or rabbit IgG isotype control at 1:100 dilution in Leica Primary Antibody diluent for 30 min at RT. Leica Bond Polymer and DAB Refine solutions were sequentially added for antibody detection. Slides were counterstained with hematoxylin before dehydration, mounting, and whole slide digital imaging (Hamamatsu NanoZoomer).

#### Statistics.

Means of more than two groups of data were compared using one-way analysis of variance (ANOVA) for analysis of one independent variable followed by Tukey’s honestly significant difference (HSD) post hoc test. We used Student’s *t* test for comparison of paired parametric data and Mann–Whitney’s *U* test for nonparametric data. All tests were two-tailed and *P* values ≤ 0.05 were considered significant. Statistical analysis was performed using GraphPad Prism for Macintosh version 8.0. All experiments were repeated with a minimum of three biological replicates and summary statistics are presented as means ± SE.

## RESULTS

### Pericytes Are Highly Enriched in Immune and Inflammatory Transcriptional Programs at Baseline and after Injury

**Figure 1.**
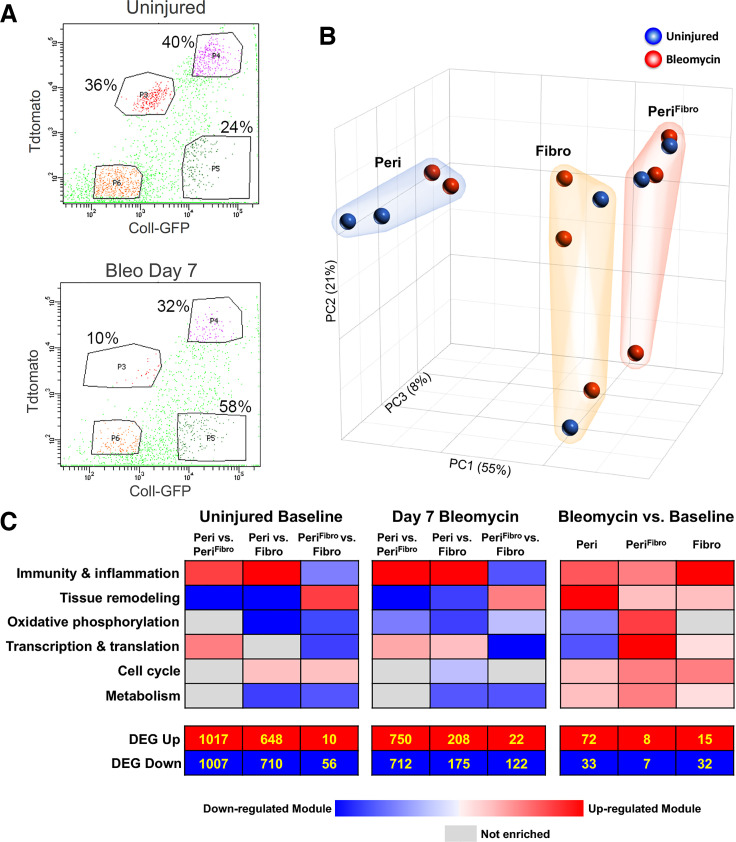
*A*: illustration of gates for FACS isolation the three populations: P3 (Peri), P4 (Peri^Fibro^), and P5 (Fibro). *B*: three-dimensional representation of PCA on the uninjured (blue) and bleomycin-injured (red) populations. Peri^Fibro^ and Fibro populations are more closely related to each other than to Peri, both with and without injury. *C*: composite figure summarizing the gene expression profiling of three stromal subpopulations (Peri, Peri^Fibro^, Fibro) at baseline (*left*), at *day 7* after bleomycin (*middle*), and comparing the populations at *day 7* bleomcyin to their respective uninjured baselines. Differential expression patterns in functional categories are displayed using a heatmap (red: upregulated, blue: downregulated). *Left*: pairwise comparisons at uninjured baseline: Peri vs. Peri^Fibro^, Peri vs. Fibro, Peri^Fibro^ vs. Fibro. The Peri population is enriched in processes involved in immunity and inflammation, whereas Fibro is enriched in processes involved in remodeling relative to Peri. *Middle*: pairwise comparisons at *day 7* after bleomycin: Peri vs. Peri^Fibro^, Peri vs. Fibro, Peri^Fibro^ vs. Fibro. *Right*: pairwise comparisons between bleomycin-injured populations at *d7* with their respective uninjured baselines. The Peri population highly upregulated programs in remodeling in injured lungs compared with uninjured baseline. *Bottom*: number of differentially expressed genes (DEG) that are up- and downregulated. *d7*, *day 7*; PCA, principal component analysis.

We previously demonstrated that stromal cell subsets with distinct transcriptional programs can be identified by *Foxd1*-lineage cells without *Coll-GFP* expression (Peri), *Foxd1-*lineage cells with *Coll-GFP* expression (Peri^Fibro^), and non-*Foxd1*-lineage cells with *Coll-GFP* expression (Fibro) (12). In this study, we analyzed global transcriptional changes in sorted populations from triple transgenic reporter animal *Foxd1-Cre;Rosa26-Tdtomato;Coll-GFP* at baseline and at *day 7* after bleomycin (Fig. 1, *A*–*C*, and GSEA table in Supplemental Table S1, all Supplemental material is available at: https://doi.org/10.6084/m9.figshare.18345221). The three populations of interest were identified by FACS gates P3 (Peri), P4 (Peri^Fibro^), and P5 (Fibro; Fig. 1*A*). Periycte-derived population (Peri and Peri^fibro^) comprised ∼76% of the cells collected versus 24% Fibro in the injured mice. By contrast, 42% of the cells collected were pericyte-derived (Peri and Peri^fibro^) and 58% were Fibro from *day 7* injured mouse lungs. Principal component analysis (PCA) of the entire gene expression data segregated the three populations, particularly Peri (Fig. 1*B*), implying that the primary driver of transcriptional variance is cell type and not exposure to bleomycin. This finding was supported by differential gene expression analysis that showed much larger number of differentially expressed genes (DEGs) between Peri versus Peri^Fibro^ and Peri versus Fibro at baseline or after bleomycin injury (Fig. 1*C*). We had previously demonstrated that stromal cell subsets with distinct gene expression profiles can be identified by *Foxd1*-lineage cells without *Coll-GFP* expression (Peri), *Foxd1-*lineage cells with *Coll-GFP* expression (Peri^Fibro^), and non-*Foxd1*-lineage cells with *Coll-GFP* expression (Fibro) (12). Using GSEA, we again demonstrated that the Peri population is highly enriched in immune and inflammatory transcriptional programs compared with Peri^Fibro^ and Fibro populations in the uninjured mice (Fig. 1*C*, Uninjured Baseline), whereas remodeling processes were highly underrepresented in Peri relative to Peri^Fibro^ and Fibro. This transcriptional pattern remained preserved at *day 7* after bleomycin, indicating persistent differential enrichment of immuno-inflammatory programs in FoxD1-derived^+^/Coll-GFP^−^ population (Peri). Interestingly, the Peri^Fibro^ cells were highly enriched in tissue remodeling programs compared with Peri and Fibro populations, both at baseline and at *d7* after bleomycin, suggesting this subset of pericytes may have high fibrogenic potential in injury. When we compared the three populations at *d7* bleomcyin to their uninjured states, the Peri population significantly upregulated remodeling programs after bleomycin injury (Fig. 1*C*, bleomycin vs. baseline), lending support to the idea that these cells might also be important contributors to the fibrotic process. We conducted a network analysis of the most highly upregulated genes using gene product interaction (Ingenuity Pathway Analysis, IPA) in the injured Peri population to further characterize the interaction between inflammatory and profibrotic genes in this subpopulation (Supplemental Fig. S3). *Spp1*, *Timp1*, *Mmp14*, *Icam1*, and *Areg* are some the genes that comprise nodes linking inflammatory and fibrotic transcriptional responses in pericytes. Based on our understanding of the transcriptional responses of the Peri and Peri^Fibro^ populations at baseline and after injury, we speculated that pericytes may have functional roles in fibrosis and/or inflammation. To further examine the dominant function of pericytes in lung injury, we conducted pericyte ablation experiments with bleomycin-induced lung injury.

### Early Ablation of Pericytes Alters Inflammation, but Not Fibrosis

**Figure 2.**
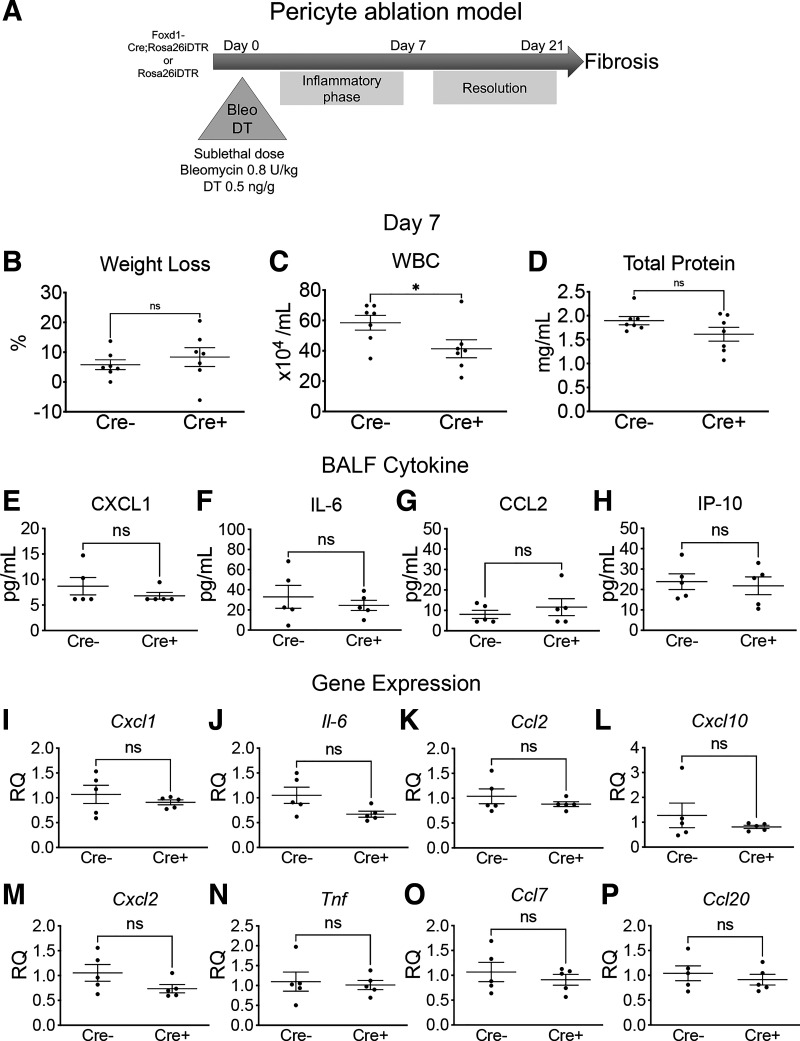
*A*: schematic of early ablation model with coadministration of bleomycin and DT. *B*: weight loss at *d7* in DT-insensitive (Cre^−^) and DT-sensitive (Cre^+^) animals. *C* and *D*: total white blood cell (WBC) count and total protein in BALF at *d7* (means ± SE, *n* = 7/group, *P < 0.05). *E*–*H*: select inflammatory cytokines in BALF by Luminex assay. *I*–*P*: expression of select inflammatory genes by qPCR of whole lung RNA (means ± SE, *n* = 5/group). BALF, bronchoalveolar lavage fluid; *d7*, *day 7*; DT, diphtheria toxin; ns, not significant.

To test the effect of early pericyte ablation on lung injury and fibrosis, we coadministered bleomycin and DT by intratracheal instillation in *Foxd1-Cre;Rosa26-iDTR* (Cre^+^) and the DT insensitive littermates *Rosa26-iDTR* (Cre^−^). DT administration at low dose (0.5 ng/g) alone did not lead to inflammatory changes at *day 2* (*d2*) and *day 7* (*d7*) following DT administration but was able to achieve a 40%–50% reduction in pericytes, defined by PDGFRβ expression by flow cytometry, at *d7* (13). When combined with bleomycin administration, early pericyte ablation decreased acute inflammation in the lung as measured by total white blood cells (WBCs) and protein 2 days following bleomycin administration (10). We evaluated whether this effect persisted to *d7* after bleomycin in the early pericyte ablation model (Fig. 2*A*) before evaluating its effect on fibrosis development at *d21*. We observed decreased WBCs in BALF of Cre^+^ mice versus Cre^−^ mice at *d7* without differences in total protein or weight loss (Fig. 2, *B*–*D*).

Pericyte ablation leads to decreased BALF total protein and WBCs, as well as decreased select proinflammatory cytokines in the BALF 2 days after pericyte ablation in the bleomycin injury model (10). To further characterize the effect of early pericyte ablation on the acute inflammatory response of the bleomycin model, we assessed select cytokines and chemokines in BALF at *d7* in the early ablation model. By *day 7*, cytokines and chemokines were low to undetectable in the BALF without significant differences between the two groups (Fig. 2, *E*–*H*). Expression of select proinflammatory genes at *d7*, evaluated by qPCR of whole lung RNA, was not significantly different between Cre^−^ and Cre^+^ animals, although there was a nonsignificant decrease in *Il-6* expression in the Cre^+^ group (*P* = 0.060; Fig. 2, *I*–*P*).

**Figure 3.**
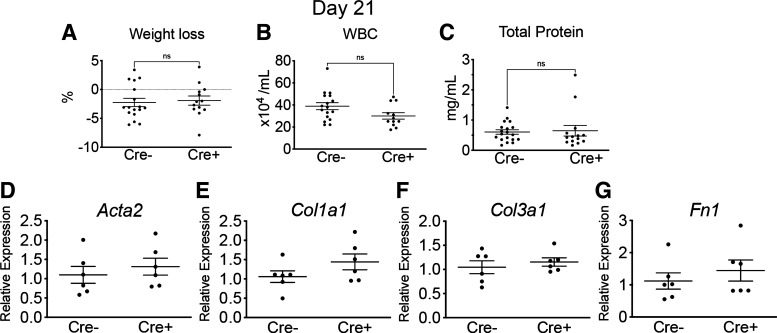
*A*–*C*: weight loss, WBCs, and total protein in BALF at *d21* (means ± SE, *n* ≥ 12/group). *D*–*G*: qPCR of RNA isolated from right lung at bleomcycin *d21*. Genes associated with fibrosis including smooth muscle actin-α (*Acta2*), collagen type 1, α1 (*Col1a1*), and fibronectin (*Fn1*) are shown (means ± SE, *n* = 5/group). BALF, bronchoalveolar lavage fluid; *d21*, *day 21*; ns, not significant; WBCs, white blood cells.

**Figure 4.**
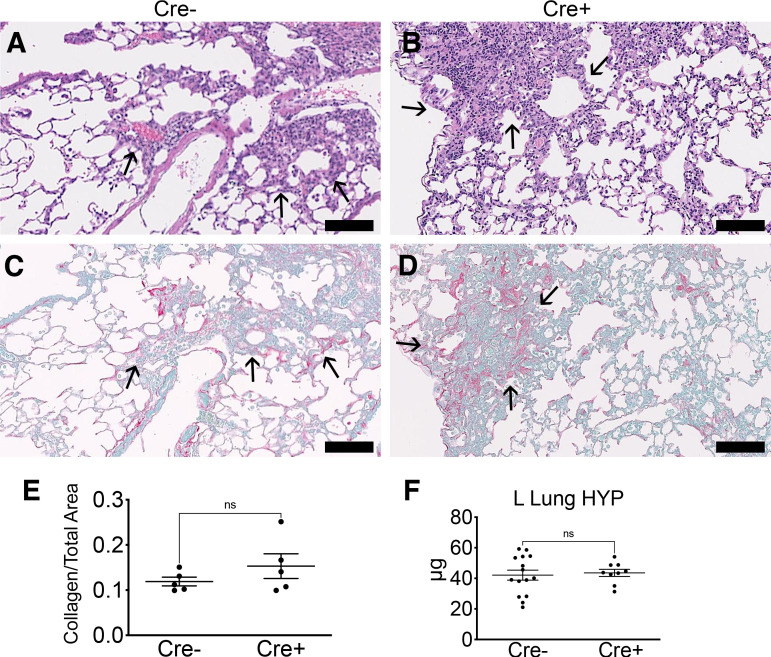
*A*–*D*: representative H&E (*A* and *B*) and picrosirius red/fast green (*C* and *D*) images from right lungs of Cre^−^ and Cre^+^ mice at bleomycin *d21*. Scale bar = 100 µm. *E*: quantitative analysis of picrosirius red positive/total lung area from whole slide scanned images. Graphs depict the area of collagen staining (picrosirius red^+^) as a ratio of total lung area (means ± SE, *n* = 5/group). *F*: hydroxyproline measurement in left lung (means ± SE, *n* ≥ 9/group). *d21*, *day 21*; H&E, hematoxylin and eosin; ns, not significant.

Next, we evaluated the effect of early ablation on markers of fibrosis through histological, biochemical, and transcriptional assessment. Without bleomycin, DT alone did not lead to changes in inflammatory response (as measured by BALF WBCs) or fibrosis (as measured by left lung hydroxyproline) at *d21* (Supplemental Fig. S1*A*). With bleomycin-induced lung injury, mice experienced similar weight changes at *day 21* (Fig. 3*A*). Evaluation of BALF revealed no significant difference in WBCs and total protein, although there was a nonsignificant trend toward lower WBCs in Cre^+^ mice at *day 21* (*P* = 0.06; Fig. 3, *B* and *C*). Quantitative assessment of fibrosis-associated genes by qPCR in lung homogenate from *d21* did not reveal significant differences in the expression of smooth muscle actin-α (*Acta2*), collagen type 1, α1 (*Col1a1*), and fibronectin (*Fn1*; Fig. 3, *D*–*G*). Representative images from Cre^+^ and Cre^−^ lungs at *d21* are presented in Fig. 4, *A*–*D*. We analyzed collagen I staining (picrosirius red positive) and the total area of lung sections using Visiopharm software to calculate the collagen/total area ratio as quantitative histology measure. The proportion of collagen-to-total lung area in Cre^+^ animals was not significantly different compared with Cre^−^ animals (Fig. 4*E*). Furthermore, biochemical assessment of collagen content by hydroxyproline of left lungs did not reveal a significant difference between Cre^+^ and Cre^−^ animals (Fig. 4*F*). Despite differences observed in markers of acute inflammation with early pericyte ablation, measures of fibrosis were not significantly altered.

### Late Pericyte Ablation Does Not Alter Lung Fibrosis

**Figure 5.**
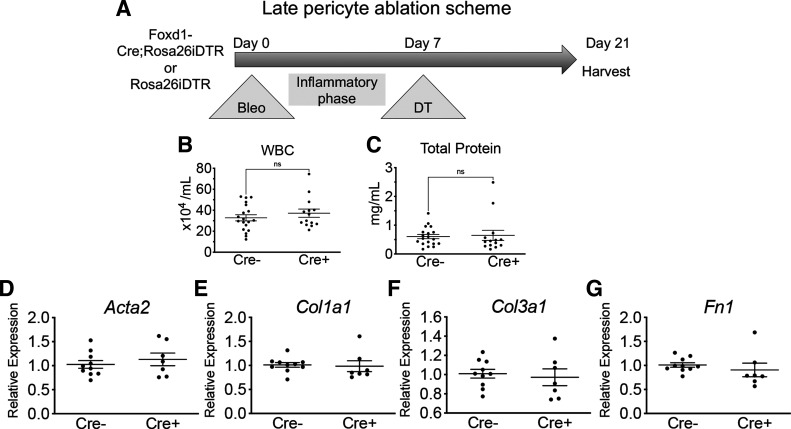
*A*: schematic of late ablation model with administration of bleomycin on *d0* followed by administration of DT on *d7*. *B* and *C*: WBCs and total protein in BALF at bleomycin *d21* (means ± SE, *n* ≥ 14/group). *D–G*: qPCR of RNA isolated from right lung at bleomcycin *d21*. Genes associated with fibrosis including smooth muscle actin-α (*Acta2*), collagen type 1, α1 (*Col1a1*), and fibronectin (*Fn1*) are shown (means ± SE, *n* = 5/group). BALF, bronchoalveolar lavage fluid; *d0*, *day 0*; *d7*, *day 7*; *d21*, *day 21*; DT, diphtheria toxin; ns, not significant; WBCs, white blood cells.

**Figure 6.**
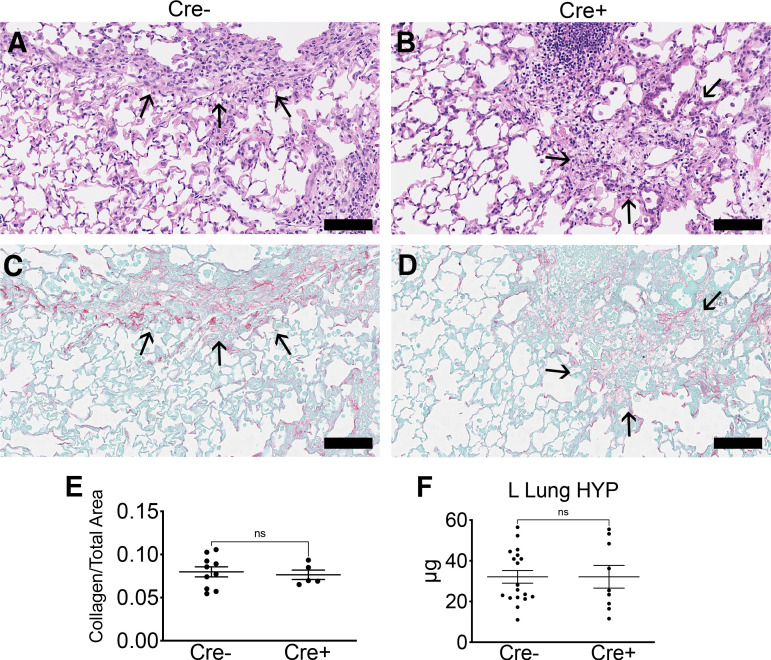
*A*–*D*: representative H&E (*A* and *B*) and picrosirius red/fast green (*C* and *D*) images from right lungs of Cre^−^ and Cre^+^ mice at bleomycin *d21*. Scale bar = 100 µm. *E*: quantitative analysis of picrosirius red positive/total lung area from whole slide scanned images. Graphs depict the area of collagen staining (picrosirius red+) as a ratio of total lung area (means ± SE, *n* ≥ 5/group). *F*: hydroxyproline measurement in left lung (means ± SE, *n* ≥ 9/group). *d21*, *day 21*; H&E, hematoxylin and eosin; ns, not significant.

As early ablation of lung pericytes altered acute inflammation, we investigated whether late ablation of lung pericytes after acute inflammation (*day 7*) affected fibrosis (Fig. 5*A*). Acute inflammation did not affect the efficiency of late ablation by DT. Cre^−^ and Cre^+^ mice injured with bleomycin were given DT at *d7* as described (Fig. 5*A*), and evaluation of ablation efficiency by flow cytometry 7 days after DT revealed greater than 50% reduction in PDGFRβ^+^ stromal cells in Cre^+^ mice (Supplemental Fig. S1, *B* and *C*), similar to the reduction seen without bleomycin injury (13). Following late ablation, BALF WBCs and total protein were similar between Cre^−^ and Cre^+^ groups at *d21* (Fig. 5, *B* and *C*). On the transcriptional level, expression of *Acta2*, *Col1a1*, *Col3a1*, and *Fn1* in whole lung was not significantly different between Cre^−^ and Cre^+^ groups at *d21* (Fig. 5, *D*–*G*). Histological evaluation of the lung sections revealed no significant difference in collagen deposition in right lungs at *d21* (Fig. 6*E*). Similarly, biochemical evaluation of left lungs by hydroxyproline assay revealed no significant difference between Cre^−^ and Cre^+^ groups (Fig. 6*F*).

## DISCUSSION

The cellular origins of lung myofibroblasts and their contribution to established fibrosis in the lung remain a topic of much controversy. We previously demonstrated that *Foxd1*-derived cells are lung pericyte-like cells and have myofibroblastic potential in vitro and in vivo, as defined by αSMA expression (12). The transcriptional landscape of the Peri, Peri^Fibro^, and Fibro populations at uninjured baseline in this report is consistent with our prior study, showing enrichment of immune-related programs in the Peri population and matrix-related programs in the Peri^Fibro^ and Fibro populations (12). Interestingly, our microarray data showed that *Foxd1*-derived pericytes (Peri and Peri^Fibro^) maintained their transcriptional identities during lung injury with an immune-related population (Peri) and a highly fibrogenic population (Peri^Fibro^), although both populations upregulated remodeling programs in response to injury. We further examined the relationships between upregulated genes in the Peri population following bleomycin-induced lung injury using gene product interaction (IPA). Genes known to have mechanistic relevance in fibrosis and lung inflammation, such as *Mmp14*, *Timp1*, *Icam1*, *Areg*, and *Spp1* were central nodes networked to other upregulated genes in the injured Peri population, suggesting potential mechanistic pathways for regulation of fibrosis and inflammation in activated pericytes.

We then correlated data from the Peri population at *d7* following bleomycin with single-cell RNAseq data in pericytes from human idiopathic pulmonary fibrosis (IPF) lungs (20). We discovered common repair and profibrotic genes that are upregulated in pericytes from IPF and bleomycin-injured lungs. Some of these include *Igfbp4*, *Fbn1*, *Col5a1*, *Col18a1*, and *Scgb1a1* (Supplemental Table S2). This correlation must be interpreted with important limitations in mind. First, the data sets were generated from different methodologies: microarray assay for murine cells in our study and single-cell RNAseq for human IPF lung cells in the referenced work. Second, the microenvironment of bleomycin injured lungs at *day 7* in mice is biologically different from that of human IPF lungs where little inflammation is apparent histologically. Nevertheless, correlation of these datasets revealed that lung pericytes in murine and IPF lungs upregulate common matrix-associated genes, suggesting the relevance of this cell type in lung fibrosis. We therefore speculated that ablation of *Foxd1-*derived pericytes would attenuate fibrosis in the bleomycin model of lung injury, and potentially altering the inflammatory response.

To test this hypothesis, we leveraged a model of lung pericyte-like cell ablation developed in our laboratory to investigate the functional consequence of pericyte-like cell ablation on the bleomycin model of lung fibrosis (13). Goritz et al. (21) conducted a similar functional interrogation of pericyte contribution to scar tissue formation in spinal cords of transgenic mice. In their study, the investigators demonstrated accumulation of lineage-labeled pericytes in scar tissue of spinal cords following experimental transection of the dorsal spinal cord. Lineage-restricted genetic knockout of all *ras* genes in pericytes (rendering them incapable of proliferation) before experimental transection led to decreased scar formation or failure to close the transected wounds, suggesting pericytes are functionally necessary for scar formation in the spinal cord. To our knowledge, a similar functional assessment of pericytes in lung fibrosis has never been published.

We previously demonstrated that lung pericyte ablation attenuates WBCs and total protein in BALF 48 h following bleomycin (10). Select cytokines in the BALF are also decreased, including IL-6 and MCP-1 (10). In the current study, attenuation of WBCs in BALF persisted to *d7* after bleomycin injury in pericyte ablated mice, although no changes in select cytokines or total protein in the BALF were seen. We considered the possibility that DT administration ablated leukocytes in the alveolar space in this model, leading to the observed decrease in BALF WBCs in Cre^+^ mice at *d7*. However, we previously showed that *Foxd1* expression is restricted to stromal cell progenitors (i.e., tdTomato^+^ cells in *Foxd1-Cre;Rosa26-tdTomato* mice do not express CD45, CD31, or CD326) and is not reexpressed in adulthood or injury (12). Furthermore, administration of low-dose DT (0.5 ng/g) alone to *Foxd1-Cre;Rosa26-iDTR* mice did not alter BALF WBCs between Cre^−^ and Cre^+^ animals at *day 2* and *day 7* (13) or at *day 21* (Supplemental Fig. S1*A*). Collectively, these data suggest leukocyte ablation in this transgenic model is an unlikely explanation for the observed attenuation of BALF WBCs in pericyte-ablated, bleomycin-injured mice. Rather, we speculate pericytes may play a role in the early inflammatory response in the bleomycin injury model, although the precise mechanism of pericyte involvement in inflammation remains to be elucidated. Potential mechanisms include directed trafficking of leukocytes across the interstitium via matrix remodeling and upregulation of leukocyte adhesion molecules, similar to what had been shown in cremaster muscle (22), and upregulation of chemokines (23). Indeed, we have previously demonstrated that lung pericyte-like cells upregulate proinflammatory processes similar to what has been shown in other organs, including adhesion molecules (e.g., VCAM and ICAM) and cytokines/chemokines (e.g., MCP-1, CXCL1, and IL-6) (10, 11). Although the regulation of lung inflammation is beyond the scope of this report, these findings collectively support further investigation into the novel mechanisms by which pericytes direct leukocyte response in the lung.

Since early pericyte ablation altered acute inflammation in the bleomycin injury model, we tested the effect of lung pericyte ablation on lung fibrosis using two different schemes: first in early ablation, and second in late ablation, when lung pericytes were eliminated after the acute inflammatory phase to bypass the effect of pericyte ablation on lung inflammation. To our surprise, selective ablation of lung pericyte-like cells at the early and at the late timepoints did not affect lung fibrosis by histological or biochemical measures. There may be several possible explanations for these results.

First, our *Foxd1-Cre;Rosa26-iDTR* model ablates ∼40% of PDGFRβ^+^ cells by oropharyngeal aspiration (13). This partial ablation may not be sufficient to result in measurable alterations to fibrosis in our experimental model. However, this degree of partial ablation was sufficient to alter acute inflammation at *d7*, and its effect on fibrosis should be detectable if pericyte-like cells were highly fibrogenic.

Rather, we speculate functional redundancy in stromal subpopulations to be the more likely explanation for our findings. In our triple transgenic reporter model (*Foxd1-Cre;Rosa26Tdtomato;CollGFP*), the Fibro population defined by GFP^+^ Tdtomato^−^ (Fig. 1*A*) is enriched for matrix-related transcriptional programs and functions at baseline (12). This population may sufficiently compensate for the ablated lung pericytes during fibrosis. This is further supported by observations of PDGFRβ immunostaining in fibrotic foci of pericyte-ablated lungs. We observed diminished PDGFRβ staining with preserved cellularity of expanded lung interstitium, in contrast to fibrotic foci in nonablated lungs where PDGFRβ staining is intense (Supplemental Fig. S2). In this context, pericyte-like cells may contribute to but are not necessary for scar formation in the lung. Moreover, other cell types that contribute to fibrogenesis in the lung, including circulating fibrocytes, epithelial cells (or transitional phenotypes that display partial mesenchymal and epithelial features), and immune mediators, are unaffected in this model and may continue to support fibrogenesis in highly fibrogenic stromal subpopulations (1–4, 6, 8, 24–26).

Alternatively, αSMA^+^ myofibroblasts may have heterogeneous capacity to produce extracellular matrix (ECM) proteins, and their fibrogenic potential may be informed by their progenitor lineage. Although we showed previously that *Foxd1*-derived cells can express αSMA with injury, their ability to deposit ECM proteins is unknown. Stromal cells derived from non-*Foxd1*-lineage populations may be responsible for the bulk of collagen deposition in the bleomycin model (e.g., αSMA^+^ GFP^+^ Tdtomato^−^). Therefore, selective ablation of *Foxd1*-derived myofibroblasts would not result in attenuated fibrosis as measured by collagen I deposition. The notion that myofibroblasts derived from different progenitors exhibit unique transcriptional signatures and functions was demonstrated in a recent study of skin myofibroblasts (27). Whether progenitor lineages of myofibroblasts in the lung also inform their molecular signatures and functions in the fibrotic scar warrants further investigation. In addition, defining myofibroblasts by αSMA^+^ may not adequately capture the totality of matrix-producing cells in fibrosis. There may be highly fibrogenic, αSMA^−^ stromal cells in lung injury that are unaffected by pericyte-like cell ablation, leading to our observations in this study.

In summary, pericytes have been implicated in organ fibrosis in many organs including the central nervous system, kidneys, and the lung. More recent advances in lineage-tracing models and transcriptomic data analysis reveal pericyte-like cells as important myofibroblast progenitors (21, 28). Despite these findings, studies establishing their functional role in disease pathogenesis have been limited. In this study, we demonstrate that pericyte ablation does not alter lung fibrosis, despite our previous work revealing this population as important progenitors of αSMA^+^ myofibroblasts (12). Our results highlight the biological complexity in lung fibrosis and the need for functional studies to understand the fibrogenic role of myofibroblast precursor populations in organ fibrosis.

## DATA AVAILABILITY

The data that support the findings of this study are available in Gene Expression Omnibus at https://www.ncbi.nlm.nih.gov/geo/, GSE184761.

## SUPPLEMENTAL DATA

10.6084/m9.figshare.18345221 Supplemental Figs. S1–S3 and Supplemental Table S1: https://doi.org/10.6084/m9.figshare.18345221.

## GRANTS

Funding for this work was provided by National Institutes of Health (NIH) Grants R01HL122895 (to W.A.A.), K08HL127075 (to C.F.H.), R03HL155075-01A1 (to C.F.H.), and R01HL133751 (to L.M.S.). This work was supported in part by the Cell Evaluation & Therapy Shared Resource, Hollings Cancer Center, Medical University of South Carolina (P30 CA138313, to L.M.S.).

## DISCLOSURES

No conflicts of interest, financial or otherwise, are declared by the authors.

## AUTHOR CONTRIBUTIONS

C.F.H., C.L.W., S.A.G., W.A.A., and L.M.S. conceived and designed research; C.F.H. and Y.-H.C. performed experiments; C.F.H., C.L.W., Y.-H.C., and S.A.G. analyzed data; C.F.H., C.L.W., Y.-H.C., W.C.L., S.A.G., and W.A.A. interpreted results of experiments; C.F.H. prepared figures; C.F.H. drafted manuscript; C.F.H., W.C.L., S.A.G., W.A.A., and L.M.S. edited and revised manuscript; C.F.H., C.L.W., Y.-H.C., W.C.L., S.A.G., W.A.A., and L.M.S. approved final version of manuscript.
